# Supplementary material for: Cost-effectiveness of maintaining an active hospital microbiology laboratory service in Timor-Leste
Source: Lancet Reg Health Southeast Asia. 2025 Apr 30;36:100582. doi: 10.1016/j.lansea.2025.100582 (PMC12076793; doi:10.1016/j.lansea.2025.100582)

**Supplementary appendix**

Cherry Lim^1,2^, Myo Maung Maung Swe^1^, Angela Devine^3,4^, Tessa Oakley^5^, Karen Champlin^5^, Pyae Sone OO^1^, Nevio Sarmento^5^, Ismael Da Costa Barreto^6^, Rodney C Givney^5^, Jennifer Yan^5^, Joshua R Francis^5^, Ben S Cooper^1,2^

^1^Centre for Global Health Research, Nuffield Department of Medicine, University of Oxford, Oxford, United Kingdom; ^2^Mahidol Oxford Tropical Medicine Research Unit, Faculty of Tropical Medicine, Mahidol University, Bangkok, Thailand; ^3^Global and Tropical Health Division, Menzies School of Health Research, Charles Darwin University, Darwin, Australia; ^4^Centre for Health Policy, Melbourne School of Population and Global Health, The University of Melbourne, Melbourne, Victoria, Australia; ^5^Global and Tropical Health Division, Menzies School of Health Research, Charles Darwin University, Dili, Timor-Leste; ^6^National Institute of Public Health, Timor-Leste

Table of Contents

[Appendix Text 3](#_Toc194135175)

[Appendix Figure 1. Decision tree 5](#_Toc194135176)

[Appendix 1. CHEERS 2022 Checklist 6](#_Toc194135177)

[Appendix 2. Expert elicitation study to inform probabilities relating to the patient’s condition and to antibiotic prescription behaviour in the absence of microbiological testing service 8](#_Toc194135178)

[Appendix 3. Model input values on transition probability and justifications. 11](#_Toc194135179)

[Appendix 4. Model input values on costs and source of data 24](#_Toc194135180)

[Appendix 5. Model input values on treatment outcome 27](#_Toc194135181)

[Appendix 6. Estimated incremental costs and disability-adjusted life years (DALYs) averted per 1,000 hospitalised paediatric patients from maintaining a microbiology laboratory compared to no microbiological testing under the baseline scenario. Costs are in 2022 US dollars. 32](#_Toc194135182)

[Appendix 7. Results of sensitivity analyses assuming two sets of blood samples (four bottles) for hospitalised adult patients with suspected primary bacteraemia. 32](#_Toc194135183)

[Appendix 8. Results of sensitivity analyses varying the cost of maintaining an active microbiology laboratory service per sample performed for hospitalised adult patients with suspected primary bacteraemia. 33](#_Toc194135184)

# Appendix Text

**Methods- Decision tree model**

The observed microbiology testing result, in the active microbiology laboratory testing system arm, may not always represent the true infection status because the sensitivity and specificity of microbiology testing is not 100%, therefore the true infection status of a patient is assumed to be imperfectly observed. In the active microbiology laboratory testing system arm, the microbiology testing results are assumed to depend on the true infection status of the patient and the performance characteristics of the testing procedures at the local microbiology laboratory.

We assume each patient undergoes an antibiotic treatment adjustment after three days of empirical treatment that depends on which comparison arm they are under (Figure 1). For patients in the active microbiology laboratory arm, in which blood culture was performed, antibiotic treatment changes would depend on the results of blood culture.

For patients in the no microbiology testing service arm, antibiotic treatment changes would depend on changes in clinical condition. We conducted an expert elicitation exercise to determine the transition probabilities of: (1) changes in clinical condition after 3 days of empiric treatment in patients with confirmed bacterial bloodstream infection, and (2) changes in antibiotic treatment based on different clinical conditions.

We consider three possible changes to the antibiotic treatment received following initial empirical treatment:

- *stepping up*, which we define as switching to a broader spectrum or last-resort antibiotic (or antibiotic combination) based on AWaRe category, which classifies antibiotics based on their spectrum of activity and potential for resistance.^10^ An example of *stepping up* would be changing from ampicillin and gentamicin, or ceftriaxone and gentamicin, to meropenem and vancomycin.
- *stepping down*, which we define as switching to a narrower spectrum antibiotic (or antibiotic combination) or to an oral antibiotic. Examples of *stepping down* include changing from meropenem and vancomycin to ceftriaxone and gentamicin; or changing from ceftriaxone and gentamicin, to an oral augmentin.
- *no change*, which we define as patients continuing to receive the same antibiotic treatment.

# Appendix Figure 1. Decision tree

Decision tree to estimate the cost-effectiveness of an active microbiology laboratory surveillance system compared to no microbiological testing. Squares indicate decision nodes, circles indicate chance nodes, and triangles indicate the end point. Collapsed branches of the tree are indicated by “[+]” and represent repeats of those shown at corresponding nodes on other branches of the tree.

# Appendix 1. CHEERS 2022 Checklist

| **Topic** | **No.** | **Item** | **Location where item is reported** |
| --- | --- | --- | --- |
| **Title** |  |  |  |
|  | 1 | Is an active hospital microbiology laboratory cost-effective in a resource-limited setting? - a case study from Timor-Leste. | Title, Page 1 |
| **Abstract** |  |  |  |
|  | 2 | Provide a structured summary that highlights context, key methods, results, and alternative analyses. | Abstract, Page 2 |
| **Introduction** |  |  |  |
| **Background and objectives** | 3 | Give the context for the study, the study question, and its practical relevance for decision making in policy or practice. | Introduction, Page 4 |
| **Methods** |  |  |  |
| **Health economic analysis plan** | 4 | Indicate whether a health economic analysis plan was developed and where available. | Methods, Page 5 |
| **Study population** | 5 | Describe characteristics of the study population (such as age range, demographics, socioeconomic, or clinical characteristics). | Methods, Page 8-10 |
| **Setting and location** | 6 | Provide relevant contextual information that may influence findings. | Methods, Page 8-10 |
| **Comparators** | 7 | Describe the interventions or strategies being compared and why chosen. | Methods, Page 5 |
| **Perspective** | 8 | State the perspective(s) adopted by the study and why chosen. | Methods, Page 5 |
| **Time horizon** | 9 | State the time horizon for the study and why appropriate. | Methods, Page 9 |
| **Discount rate** | 10 | Report the discount rate(s) and reason chosen. | Methods, Page 9 |
| **Selection of outcomes** | 11 | Describe what outcomes were used as the measure(s) of benefit(s) and harm(s). | Methods, Page 10 |
| **Measurement of outcomes** | 12 | Describe how outcomes used to capture benefit(s) and harm(s) were measured. | Methods, Page 10 |
| **Valuation of outcomes** | 13 | Describe the population and methods used to measure and value outcomes. | Methods, Page 10-11 |
| **Measurement and valuation of resources and costs** | 14 | Describe how costs were valued. | Methods, Page 9 |
| **Currency, price date, and conversion** | 15 | Report the dates of the estimated resource quantities and unit costs, plus the currency and year of conversion. | Methods, Page 9-10 |
| **Rationale and description of model** | 16 | If modelling is used, describe in detail and why used. Report if the model is publicly available and where it can be accessed. | Methods, Page 9 |
| **Analytics and assumptions** | 17 | Describe any methods for analysing or statistically transforming data, any extrapolation methods, and approaches for validating any model used. | Methods, Page 9-13 |
| **Characterising heterogeneity** | 18 | Describe any methods used for estimating how the results of the study vary for subgroups. | Methods, Page 12 |
| **Characterising distributional effects** | 19 | Describe how impacts are distributed across different individuals or adjustments made to reflect priority populations. | Methods, Page 13 |
| **Characterising uncertainty** | 20 | Describe methods to characterise any sources of uncertainty in the analysis. | Methods, Page 13 |
| **Approach to engagement with patients and others affected by the study** | 21 | Describe any approaches to engage patients or service recipients, the general public, communities, or stakeholders (such as clinicians or payers) in the design of the study. | Appendix 1 |
| **Results** |  |  |  |
| **Study parameters** | 22 | Report all analytic inputs (such as values, ranges, references) including uncertainty or distributional assumptions. | Results, Page 13 |
| **Summary of main results** | 23 | Report the mean values for the main categories of costs and outcomes of interest and summarise them in the most appropriate overall measure. | Results, Page 13 |
| **Effect of uncertainty** | 24 | Describe how uncertainty about analytic judgments, inputs, or projections affect findings. Report the effect of choice of discount rate and time horizon, if applicable. | Results, Page 13-19 |
| **Effect of engagement with patients and others affected by the study** | 25 | Report on any difference patient/service recipient, general public, community, or stakeholder involvement made to the approach or findings of the study | Not reported |
| **Discussion** |  |  |  |
| **Study findings, limitations, generalisability, and current knowledge** | 26 | Report key findings, limitations, ethical or equity considerations not captured, and how these could affect patients, policy, or practice. | Discussion |
| **Other relevant information** |  |  |  |
| **Source of funding** | 27 | Describe how the study was funded and any role of the funder in the identification, design, conduct, and reporting of the analysis | End of manuscript |
| **Conflicts of interest** | 28 | Report authors conflicts of interest according to journal or International Committee of Medical Journal Editors requirements. | End of manuscript |

*From:* Husereau D, Drummond M, Augustovski F, et al. Consolidated Health Economic Evaluation Reporting Standards 2022 (CHEERS 2022) Explanation and Elaboration: A Report of the ISPOR CHEERS II Good Practices Task Force. Value Health 2022;25. <doi:10.1016/j.jval.2021.10.008>

# Appendix 2. Expert elicitation study to inform probabilities relating to the patient’s condition and to antibiotic prescription behaviour in the absence of microbiological testing service

**Methods**

We performed a structured expert elicitation exercise to generate parameter values for the decision tree. As electronic clinical data about the progress of a patient's clinical condition was not readily accessible, we consulted five experts from the hospitals about the probability of observing clinical changes in a hospitalised patient who was suspected of primary bloodstream infection after different empirical antibiotic treatments.

Five experts were selected based on four inclusion criteria:

[1] have experience and qualification in prescribing antibiotics to paediatrics or adult patients

[2] with at least two years of clinical experience in hospital(s) in Timor-Leste

[3] have experience in treating at least 10 patients with sepsis or bloodstream infections within the past year (2022)

[4] have good understanding on empirical antibiotic treatment and have experience in revising treatment regimen (including type, dose, frequency and route) based on changes in patient’s clinical conditions.

The structured expert elicitation was performed based on recommendation of Bojke et al (2021). In brief, five experts were interviewed independently and in each interview an initial training and then followed by an elicitation session were performed. There were in total 16 questions asked, of which 4 were seed questions and were used to weigh the answers of the experts during the analysis. For each elicited question, data collected from all five experts were combined using a linear pool, then the quantiles (median, 25th quantile, and 75th quantile) were calculated from the pooled cumulative distribution. Analysis code is available in <https://github.com/Cherrylim128/CEA_maintain_active_microbiology/tree/main>.

**The question asked were:**

In hospitalized patients with suspected bloodstream infection (SBI) in Timor-Leste, what proportion of patients (clinical conditions) are likely to deteriorate, unchange or improve?

(4 questions)

1. In patients with true bacterial infection after receiving a 3-day of empirical treatment with ceftriaxone plus gentamicin?
2. In patients with true bacterial infection after receiving a 3-day of empirical treatment with ampicillin plus gentamicin?
3. In patients with true bacterial infection after receiving a 3-day of empirical treatment with meropenem plus vancomycin?
4. In patients without bacterial infection (after a 3-day empirical antibiotic treatment)?

For patients empirically treated with **ceftriaxone plus gentamicin.** (3 questions)

1. What proportion of patients need step-up antibiotic treatment in patients whose clinical condition deteriorates after receiving a 3-day of empirical treatment with ceftriaxone plus gentamicin?
2. What proportion of patients need step-up antibiotic treatment in patients whose clinical condition remains unchanged after receiving a 3-day of empirical treatment with ceftriaxone plus gentamicin?
3. What proportion of patients need step-down antibiotic treatment in patients whose clinical condition improves after a 3-day of empirical treatment with ceftriaxone plus gentamicin?

For patients empirically treated with **ampicillin plus gentamicin** (3 questions)

1. What proportion of patients need step-up antibiotic treatment in patients whose clinical condition deteriorates after receiving a 3-day of empirical treatment with ampicillin plus gentamicin?
2. What proportion of patients need step-up antibiotic treatment in patients whose clinical condition remains unchanged after receiving a 3-day of empirical treatment with ampicillin plus gentamicin?
3. What proportion of patients need step-down antibiotic treatment in patients whose clinical condition improves after a 3-day of empirical treatment with ampicillin plus gentamicin?

For patients empirically treated with **meropenem plus vancomycin** (3 questions)

1. What proportion of patients need step-up antibiotic treatment in patients whose clinical condition deteriorates after receiving a 3-day of empirical treatment with meropenem plus vancomycin?
2. What proportion of patients need step-up antibiotic treatment in patients whose clinical condition remains unchanged after receiving a 3-day of empirical treatment with meropenem plus vancomycin?
3. What proportion of patients need step-down antibiotic treatment in patients whose clinical condition improves after a 3-day of empirical treatment with meropenem plus vancomycin?

**Results** **Table. Estimated probabilities from independent interviews with five experts and analysis was performed using a weighted linear pool.**

|  | **Given if patients had bacterial infection (a latent unobserved event when empirical antibiotic treatment) and no microbiological test performed** | | | **No bacterial infection** |
| --- | --- | --- | --- | --- |
|  | **Ceftriaxone combined with gentamicin** | **Ampicillin combined with gentamicin** | **Meropenem combined with vancomycin** |  |
| **Deteriorated** | 0.24 | 0.43 | 0.07 | 0.07 |
| **Improved** | 0.60 | 0.42 | 0.87 | 0.37 |
| **Unchanged** | 0.16 | 0.15 | 0.06 | 0.56 |
| **Step-up when deteriorated** | 0.70 | 0.96 | 0.012 | ND |
| **Step-up when unchanged** | 0.56 | 0.74 | 0.013 | ND |
| **Step-down when improved** | 0.22 | 0.53 | 0.20 | ND |

# Appendix 3. Model input values on transition probability and justifications.

| **Input variable** | **Baseline scenario** | **Lower value of deterministic sensitivity analysis** | **Upper value of deterministic sensitivity analysis** | **Probabilistic distribution** | **Source of data and justifications** |
| --- | --- | --- | --- | --- | --- |
| **True underlying cause of suspected bloodstream infection** |  |  |  |  | https://www.ncbi.nlm.nih.gov/labs/pmc/articles/PMC6322461 |
| Probability of that the suspected BSI case truly has a bacterial infection | 0.40 | 0.15 | 0.65 | p.infecttype ~ beta(40,60) | Microbiology data from Timor Leste hospital    Of 1937 blood cultures, 1378 reported no bacterial growth:  - Assuming 5% of contamination rate and 80% of sensitivity, then prevalence would be around 34%  - Assuming 10% of contamination rate and 60% of sensitivity, then prevalence would be around 32%    Hence, rounding the numbers up, the baseline scenario assumed the prevalence of bacterial infection among suspected BSI is 40%  Note:  - If prevalence is only 10%, then contamination rate in the microbiological testing based on the observed culture results would be as high as 79%, which is very unlikely. Hence sensitivity test was performed for a 15% prevalence as lowest limit. |
| Probability of that the suspected BSI case has a non-bacterial infection | 0.60 | 0.85 | 0.35 |  |  |
| **Performance of blood culture** |  |  |  |  |  |
| Probability of culture positive given if the suspected BSI case has a bacterial infection | 0.80 | - | - | p.bact_culture_pos ~ rbeta(12.72, 3.18) | - |
| Probability of culture negative given if the suspected BSI case has a bacterial infection | 0.20 | - | - |  |  |
| Probability of culture positive given if the suspected BSI case has no bacterial infection | 0.05 | - | - | p.nobact_culture_pos ~ rbeta(5, 95) | - |
| Probability of culture negative given if the suspected BSI case has no bacterial infection | 0.95 | - | - |  |  |
| **Changes in antibiotic therapy given culture positive** |  |  |  |  | Based on WISCA using Timor blood culture data. See detail of WISCA estimation at the end of the table. |
| Probability of stepping down given if culture result is positive for when empirical antibiotic treatment was ceftriaxone combined with gentamicin  (Percentage of coverage for ceftriaxone combined with gentamicin)*1/10 | 0.061 | [1] 0.017  [2] 0.045 | [1] 0.075  [2] 0.135 | dirichlet (6.1,39,54.9) | Note:  Stepwise deterministic sensitivity analysis was performed |
| Probability of stepping up given if culture result is positive for when empirical antibiotic treatment was ceftriaxone combined with gentamicin  (1-Percentage of coverage for ceftriaxone combined with gentamicin) | 0.39 | [1] 0.280  [2] 0.356 | [1] 0.423  [2] 0.543 |  |  |
| Probability of no change given if culture result is positive for when empirical antibiotic treatment was ceftriaxone combined with gentamicin  (Percentage of coverage for ceftriaxone combined with gentamicin)*9/10 | 0.549 | [1] One minus the total of the above two values  [2] One minus the total of the above two values | [1] One minus the total of the above two values  [2] One minus the total of the above two values |  |  |
| Probability of stepping down given if culture result is positive for when empirical antibiotic treatment was ampicillin combined with gentamicin  (Percentage of coverage for ampicillin combined with gentamicin)/2 | 0.061 | [1] 0.017  [2] 0.045 | [1] 0.075  [2] 0.135 | dirichlet (61/10,39,54.9) |  |
| Probability of stepping up given if culture result is positive for when empirical antibiotic treatment was ampicillin combined with gentamicin  (1-Percentage of coverage for ampicillin combined with gentamicin) | 0.39 | [1] 0.280  [2] 0.356 | [1] 0.423  [2] 0.543 |  |  |
| Probability of no change given if culture result is positive for when empirical antibiotic treatment was ampicillin combined with gentamicin  (Percentage of coverage for ampicillin combined with gentamicin)/2 | 0.549 | [1] One minus the total of the above two values  [2] One minus the total of the above two values | [1] One minus the total of the above two values  [2] One minus the total of the above two values |  |  |
| Probability of stepping down given if culture result is positive for when empirical antibiotic treatment was meropenem combined with vancomycin  (Percentage of coverage for meropenem combined with vancomycin)/2 | 0.726 | [1] 0.26  [2] 0.34 | [1] 0.40  [2] 0.48 | dirichlet (72.6,26,1.4) |  |
| Probability of stepping up given if culture result is positive for when empirical antibiotic treatment was meropenem combined with vancomycin  (1-Percentage of coverage for meropenem combined with vancomycin) | 0.26 | [1] 0.16  [2] 0.23 | [1] 0.29  [2] 0.38 |  |  |
| Probability of no change given if culture result is positive for when empirical antibiotic treatment was meropenem combined with vancomycin  (Percentage of coverage for meropenem combined with vancomycin)/2 | 0.014 | [1] One minus the total of the above two values  [2] One minus the total of the above two values | [1] One minus the total of the above two values  [2] One minus the total of the above two values |  |  |
| **Health condition given culture result is negative (essentially no information on potential causative organism)** |  |  |  |  |  |
| Probability that patient’s health condition deteriorates given patient had ceftriaxone combined with gentamicin, **has bacterial infection** and culture result returned as negative | 0.048 | 0.39 | 0.39 | dirichlet (24/5,(100-(24/5)-16),16)) |  |
| Probability that patient’s health condition improves given patient had ceftriaxone combined with gentamicin, **has bacterial infection** and culture result returned as negative | 0.792 | [1] One minus the total of the above and below values | [1] One minus the total of the above and below values |  |  |
| Probability that patient’s health condition remains unchanged given patient had ceftriaxone combined with gentamicin, **has bacterial** infection and culture result returned as negative | 0.16 | [1] 0.061  [2] 0.305  (This was done with combination of the sensitivity analysis on  “Probability of that the suspected BSI case has a non-bacterial infection = 0.8”) | [1] 0.549  (This was done with combination of the sensitivity analysis on  “Probability of that the suspected BSI case has a non-bacterial infection = 0.8”) |  |  |
| Probability that patient’s health condition deteriorates given patient had ceftriaxone combined with gentamicin, **has no bacterial infection** and culture result returned as negative | 0.07 | [1] One minus the total of the below two values  [2] One minus the total of the below two values | [1] One minus the total of the below two values  [2] One minus the total of the below two values | dirichlet (7,37,56) |  |
| Probability that patient’s health condition improves given patient had ceftriaxone combined with gentamicin, **has no bacterial infection** and culture result returned as negative | 0.37 | [1] 0.68  [2] 0.78 | [1] 0.83  [2] 0.88 |  |  |
| Probability that patient’s health condition remains unchanged given patient had ceftriaxone combined with gentamicin, **has no bacterial** infection and culture result returned as negative | 0.56 | [1] 0.07  [2] 0.12 | [1] 0.17  [2] 0.12 |  |  |
| Probability that patient’s health condition deteriorates given patient had ampicillin combined with gentamicin, **has bacterial infection** and culture result returned as negative | 0.086 | 0.39 | 0.39 | dirichlet (43/5,(100-(43/5)-15),15) |  |
| Probability that patient’s health condition improves given patient had ampicillin combined with gentamicin, **has bacterial infection** and culture result returned as negative | 0.764 | [1] One minus the total of the above and below values | [1] One minus the total of the above and below values |  |  |
| Probability that patient’s health condition remains unchanged given patient had ampicillin combined with gentamicin, **has bacterial** infection and culture result returned as negative | 0.150 | [1] 0.061  [2] 0.305  (This was done with combination of the sensitivity analysis on  “Probability of that the suspected BSI case has a non-bacterial infection = 0.8”) | [1] 0.549  (This was done with combination of the sensitivity analysis on  “Probability of that the suspected BSI case has a non-bacterial infection = 0.8”) |  |  |
| Probability that patient’s health condition deteriorates given patient had ampicillin combined with gentamicin, has **no bacterial infection** and culture result returned as negative | 0.07 | [1] One minus the total of the below two values  [2] One minus the total of the below two values | [1] One minus the total of the below two values  [2] One minus the total of the below two values | dirichlet (7,37,56) |  |
| Probability that patient’s health condition improves given patient had ampicillin combined with gentamicin, **has no bacterial** infection and culture result returned as negative | 0.37 | [1] 0.68  [2] 0.78 | [1] 0.83  [2] 0.88 |  |  |
| Probability that patient’s health condition remains unchanged given patient had ampicillin combined with gentamicin, **has no bacterial** infection and culture result returned as negative | 0.56 | [1] 0.07  [2] 0.12 | [1] 0.17  [2] 0.12 |  |  |
| Probability that patient’s health condition deteriorates given patient had meropenem combined with vancomycin, **has bacterial** infection and culture result returned as negative | 0.007 | 0.26 | 0.26 | dirichlet(7/10,(100-(7/10)-6),6) |  |
| Probability that patient’s health condition improves given patient had meropenem combined with vancomycin, **has bacterial** infection and culture result returned as negative | 0.933 | [1] One minus the total of the above and below values | [1] One minus the total of the above and below values |  |  |
| Probability that patient’s health condition remains unchanged given patient had meropenem combined with vancomycin, **has bacterial** infection and culture result returned as negative | 0.060 | [1] 0.014  [2] 0.37  (This was done with combination of the sensitivity analysis on  “Probability of that the suspected BSI case has a non-bacterial infection = 0.8”) | [1] 0.726  (This was done with combination of the sensitivity analysis on  “Probability of that the suspected BSI case has a non-bacterial infection = 0.8”) |  |  |
| Probability that patient’s health condition deteriorates given patient had meropenem combined with vancomycin, has **no bacterial infection** and culture result returned as negative | 0.07 | [1] One minus the total of the below two values  [2] One minus the total of the below two values | [1] One minus the total of the below two values  [2] One minus the total of the below two values | dirichlet (7,37,56) |  |
| Probability that patient’s health condition improves given patient had meropenem combined with vancomycin, **has no bacterial** infection and culture result returned as negative | 0.37 | [1] 0.20  [2] 0.27 | [1] 0.33  [2] 0.43 |  |  |
| Probability that patient’s health condition remains unchanged given patient had meropenem combined with vancomycin, **has no bacterial** infection and culture result returned as negative | 0.56 | [1] 0.35  [2] 0.46 | [1] 0.54  [2] 0.57 |  |  |
| **Health condition given no culture done (essentially no information on potential causative organism)** |  |  |  |  |  |
| Probability that patient’s health condition deteriorates given patient had ceftriaxone combined with gentamicin, **has bacterial** infection and no culture done | 0.24 | [1] 0.22  [2] 0.30 | [1] 0.36  [2] 0.44 | dirichlet (24,60,16) | Based on results from the expert elicitation exercise (Appendix 2) |
| Probability that patient’s health condition improves given patient had ceftriaxone combined with gentamicin, **has bacterial** infection and no culture done | 0.60 | [1] One minus the total of the above and below values  [2] One minus the total of the above and below values | [1] One minus the total of the above and below values  [2] One minus the total of the above and below values |  |  |
| Probability that patient’s health condition remains unchanged given patient had ceftriaxone combined with gentamicin, **has bacterial** infection and no culture done | 0.16 | [1] 0.21  [2] 0.31 | [1] 0.37  [2] 0.45 |  |  |
| Probability that patient’s health condition deteriorates given patient had ceftriaxone combined with gentamicin, **has no bacterial** infection and no culture done | 0.07 | [1] One minus the total of the below two values  [2] One minus the total of the below two values | [1] One minus the total of the below two values  [2] One minus the total of the below two values | dirichlet(7, 37, 56) | Based on results from the expert elicitation exercise (Appendix 2) |
| Probability that patient’s health condition improves given patient had ceftriaxone combined with gentamicin, **has no bacterial infection** and no culture done | 0.37 | [1] 0.22  [2] 0.29 | [1] 0.36  [2] 0.47 |  |  |
| Probability that patient’s health condition remains unchanged given patient had ceftriaxone combined with gentamicin, **has no bacterial** infection and no culture done | 0.56 | [1] 0.35  [2] 0.47 | [1] 0.54  [2] 0.53 |  |  |
| Probability that patient’s health condition deteriorates given patient had ampicillin combined with gentamicin, **has bacterial infection** and no culture done | 0.43 | [1] 0.22  [2] 0.30 | [1] 0.36  [2] 0.44 | dirichlet (43,42,15) | Based on results from the expert elicitation exercise (Appendix 2) |
| Probability that patient’s health condition improves given patient had ampicillin combined with gentamicin, **has bacterial infection** and no culture done | 0.42 | [1] One minus the total of the above and below values  [2] One minus the total of the above and below values | [1] One minus the total of the above and below values  [2] One minus the total of the above and below values |  |  |
| Probability that patient’s health condition remains unchanged given patient had ampicillin combined with gentamicin, **has bacterial** infection and no culture done | 0.15 | [1] 0.21  [2] 0.31 | [1] 0.37  [2] 0.45 |  |  |
| Probability that patient’s health condition deteriorates given patient had ampicillin combined with gentamicin, **has no bacterial** infection and no culture done | 0.07 | [1] One minus the total of the below two values  [2] One minus the total of the below two values | [1] One minus the total of the below two values  [2] One minus the total of the below two values | dirichlet (7, 37, 56) | Based on results from the expert elicitation exercise (Appendix 2) |
| Probability that patient’s health condition improves given patient had ampicillin combined with gentamicin, **has no bacterial** infection and no culture done | 0.37 | [1] 0.22  [2] 0.29 | [1] 0.36  [2] 0.47 |  |  |
| Probability that patient’s health condition remains unchanged given patient had ampicillin combined with gentamicin, **has no bacterial** infection and no culture done | 0.56 | [1] 0.35  [2] 0.47 | [1] 0.54  [2] 0.53 |  |  |
| Probability that patient’s health condition deteriorates given patient had meropenem combined with vancomycin, **has bacterial** infection and no culture done | 0.07 | [1] 0.24  [2] 0.30 | [1] 0.36  [2] 0.44 | dirichlet(7, 87, 6) | Based on results from the expert elicitation exercise (Appendix 2) |
| Probability that patient’s health condition improves given patient had meropenem combined with vancomycin, **has bacterial** infection and no culture done | 0.87 | [1] One minus the total of the above and below values  [2] One minus the total of the above and below values | [1] One minus the total of the above and below values  [2] One minus the total of the above and below values |  |  |
| Probability that patient’s health condition remains unchanged given patient had meropenem combined with vancomycin, **has bacterial** infection and no culture done | 0.06 | [1] 0.25  [2] 0.31 | [1] 0.38  [2] 0.46 |  |  |
| Probability that patient’s health condition deteriorates given patient had meropenem combined with vancomycin, **has no bacterial** infection and no culture done | 0.07 | [1] One minus the total of the below two values  [2] One minus the total of the below two values | [1] One minus the total of the below two values  [2] One minus the total of the below two values | dirichlet (7, 37, 56) | Based on results from the expert elicitation exercise (Appendix 2) |
| Probability that patient’s health condition improves given patient had meropenem combined with vancomycin, **has no bacterial** infection and no culture done | 0.37 | [1] 0.20  [2] 0.30 | [1] 0.36  [2] 0.48 |  |  |
| Probability that patient’s health condition remains unchanged given patient had meropenem combined with vancomycin, **has no bacterial** infection and no culture done | 0.56 | [1] 0.38  [2] 0.48 | [1] 0.55  [2] 0.52 |  |  |
| **Changes in antibiotic therapy under different health conditions given culture result is negative** |  |  |  |  |  |
| Probability of stepping up given patient’s health condition deteriorates | 0.012 | ND | ND | beta (1.2, 98.8)  Range: 0.57-0.84  IQR: 0.67-0.73 |  |
| Probability of no change given patient’s health condition deteriorates | 0.988 | ND | ND |  |  |
| Probability of stepping down given patient’s health condition improves | 0.800 | ND | ND | beta (70,30)  Range: 0.57-0.84  IQR: 0.67-0.73 |  |
| Probability of no change given patient’s health condition improves | 0.200 | ND | ND |  |  |
| Probability of stepping up given patient’s health condition remains unchanged | 0.013 | ND | ND | beta (10,90)  Range: 0.03-0.20  IQR: 0.08-0.12 |  |
| Probability of no change given patient’s health condition remains unchanged | 0.987 | ND | ND |  |  |
| **Changes in antibiotic therapy under different health conditions given no culture was performed** |  |  |  |  |  |
| Probability of stepping up given patient’s health condition deteriorates | 0.90 | ND | ND | beta (90,10)  Range: 0.80-0.97  IQR: 0.88-0.92 |  |
| Probability of no change given patient’s health condition deteriorates | 0.10 | ND | ND |  |  |
| Probability of stepping down given patient’s health condition improves | 0.20 | ND | ND | beta (20,80)  Range: 0.09-0.32  IQR: 0.17-0.22 |  |
| Probability of no change given patient’s health condition improves | 0.80 | ND | ND |  |  |
| Probability of stepping up given patient’s health condition remains unchanged | 0.70 | ND | ND | beta (70,30)  Range: 0.57-0.84  IQR: 0.67-0.73 |  |
| Probability of no change given patient’s health condition remains unchanged | 0.30 | ND | ND |  |  |

**WISCA estimate based on local hospital microbiology data**

Model of WISCA used:


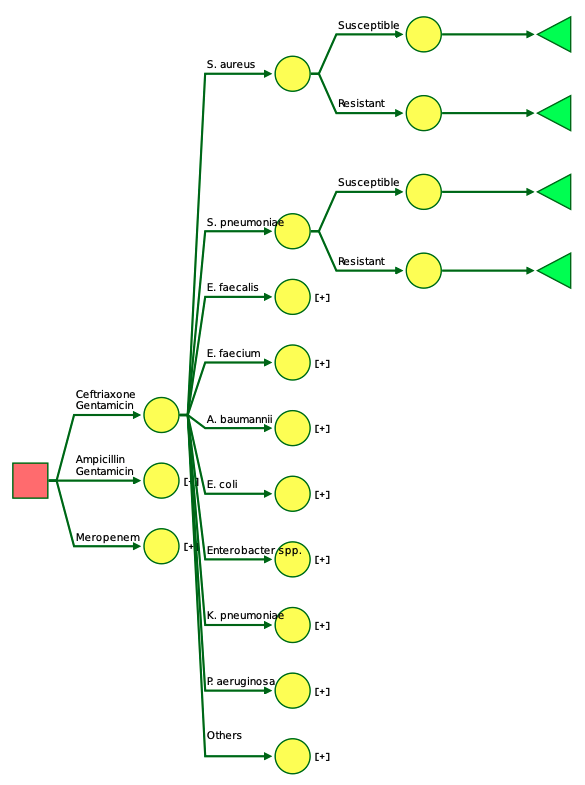


**Proportion of different pathogenic bacteria calculated using local microbiology data**

| **Pathogen** | **Proportion of the organism identified among all culture positive sample identified by the local hospital microbiology laboratory** | **Empiric antibiotic regimens** | **Proportion of susceptible to the specified empiric antibiotic among each identified organism** |
| --- | --- | --- | --- |
| ***Staphylococcus aureus*** | 9.52% | **Ceftriaxone combined with gentamicin** | 100% |
|  |  | **Ampicillin combined with gentamicin** | 100% |
|  |  | **Meropenem combined with vancomycin** | 100% |
| ***Streptococcus pneumoniae*** | 1.19% | **Ceftriaxone combined with gentamicin** | 100% |
|  |  | **Ampicillin combined with gentamicin** | 67% |
|  |  | **Meropenem combined with vancomycin** | 100% |
| ***Enterococcus faecalis*** | 4.76% | **Ceftriaxone combined with gentamicin** | 0% |
|  |  | **Ampicillin combined with gentamicin** | 53.85 |
|  |  | **Meropenem combined with vancomycin** | 0% |
| ***Enterococcus faecium*** | 1.59% | **Ceftriaxone combined with gentamicin** | 0% |
|  |  | **Ampicillin combined with gentamicin** | 0% |
|  |  | **Meropenem combined with vancomycin** | 0% |
| ***Acinetobacter baumannii*** | 8.33% | **Ceftriaxone combined with gentamicin** | 62% |
|  |  | **Ampicillin combined with gentamicin** | 38.10 |
|  |  | **Meropenem combined with vancomycin** | 57.14 |
| ***Escherichia coli*** | 5.56% | **Ceftriaxone combined with gentamicin** | 100% |
|  |  | **Ampicillin combined with gentamicin** | 100% |
|  |  | **Meropenem combined with vancomycin** | 100% |
| ***Enterobacter* spp.** | 5.56% | **Ceftriaxone combined with gentamicin** | 92.86% |
|  |  | **Ampicillin combined with gentamicin** | 92.86% |
|  |  | **Meropenem combined with vancomycin** | 100% |
| ***Klebsiella pnuemoniae*** | 15.08% | **Ceftriaxone combined with gentamicin** | 94.73% |
|  |  | **Ampicillin combined with gentamicin** | 94.73% |
|  |  | **Meropenem combined with vancomycin** | 100% |
| ***Pseudomonas aeruginosa*** | 12.30% | **Ceftriaxone combined with gentamicin** | 93.55% |
|  |  | **Ampicillin combined with gentamicin** | 93.55% |
|  |  | **Meropenem combined with vancomycin** | 87.10% |
| **Others** | 36.11% | **Ceftriaxone combined with gentamicin** | 24.18% |
|  |  | **Ampicillin combined with gentamicin** | 24.18% |
|  |  | **Meropenem combined with vancomycin** | 87.91% |

# Appendix 4. Model input values on costs and source of data

| Cost variable | Baseline scenario | Lower value of deterministic sensitivity analysis | Upper value of deterministic sensitivity analysis | Probabilistic distribution | Source of data |
| --- | --- | --- | --- | --- | --- |
| Cost of running microbiology laboratory under enhance surveillance program in one hospital per patient |  |  |  |  | Local hospital cost |
| Total cost per sample | $34.54 | $24.96 | $75 | ND |  |
| Cost one positive blood bottle |  |  |  |  | Local hospital cost |
| Total cost per positive blood bottle  - Include all consumables and staff salary | $31.90 | ND | ND | ND |  |
| Cost one negative blood bottle  - Include all consumables and staff salary |  |  |  |  | Local hospital cost |
| Total cost per negative blood bottle  - Assuming taking 2 blood bottles is a common practice in Timor hospital, hence will cost will be multiplied by 2 in the model | $5.80 | ND | ND | ND |  |
| Cost of antibiotics per day  - Assuming that each treatment will be given for 5 days, hence the cost will be multiplied by 5 in the model |  |  |  |  | **Based on Timor-Leste antibiotic guideline:**  **Benzylpenicillin 1.8g (child: 50mg/kg) IV 4 hourly for**  **4 – 6 weeks + Cloxacillin 2g (child: 50mg/kg) IV 4 hourly for 4 – 6 weeks + Gentamicin 1mg/kg (up to 80mg) IV 8 hourly for 2 weeks only** |
| Cost of (empirical treatment) ceftriaxone combined with gentamicin per day | $1.76 (Adults)  $0.64 (Children) | ND | ND | ND |  |
| Cost of (empirical treatment) ampicillin combined with gentamicin per day | $3.04 (Adults)  $1.02 (Children) | ND | ND | ND |  |
| Cost of (empirical treatment) meropenem combined with vancomycin per day | $35.16 (Adults)  $11.72 (Children) | ND | ND | ND |  |
| Cost of step-down to oral amoxicillin 1g PO TID from ceftriaxone combined with gentamicin or gentamicin combined with penicillin G per day | $0.144 (Adults)  $0.144 (Children) | ND | ND | ND |  |
| Cost of step-up to meropenem combined with vancomycin from ceftriaxone combined with gentamicin or gentamicin combined with penicillin G per day | $35.16 (Adults)  $11.72 (Children) | ND | ND | ND |  |
| Cost of step-up to colistin from meropenem combined with vancomycin per day | $100 (Adults)  $100 (Children)  In the absence of colistin  $35.16*1.5 (Adults)  $11.72*1.5 (Children) | ND | ND | ND |  |
| Cost of no change (the same cost as empirical antibiotic) per day | See above | ND | ND | ND |  |
| Cost of care per day  (not accounting for medical interventions, treatment, or devices) |  |  |  |  | **A study on intensive care of 14 countries:**  [**https://www.ncbi.nlm.nih.gov/pmc/articles/PMC3551445/**](https://www.ncbi.nlm.nih.gov/pmc/articles/PMC3551445/)  **(reports costs per capita healthcare cost)**  **See table from paper below**    [**https://www.researchgate.net/publication/228582375_Unit_costs_of_health_care_inputs_in_low_and_middle_income_regions**](https://www.researchgate.net/publication/228582375_Unit_costs_of_health_care_inputs_in_low_and_middle_income_regions)  **Cost per inpatient hospital bed day in 2001 in tertiary hospital in South Asia was estimated:**  **Low: $2.6461; high: $25.87; best: $11.61** |
| Cost of care for when clinical condition deteriorates per patient  - sum of salary of HCW and per capita healthcare cost in an ICU  ($16.19+$187) | $203.19 | $100 | $300 | lognormal(log(203.19), 0.25)  Range: $51.59-$234.29  IQR: $90.92-$129.09 |  |
| Cost of care for when clinical condition improved  - sum of salary of HCW and per capita healthcare cost in internal medicine ward  ($2.83+$11.61) | $14.44 |  |  | lognormal(log(14.44), 0.25)  Range: $6.26-$34.63  IQR: $12.40-$17.01 | Cost per bed-day for tertiary hospital in Indoneia: $47.80 in 2000  https://apps.who.int/iris/bitstream/handle/10665/42699/9241546018.pdf?sequence= |
| Cost of care for when clinical condition has no change (average of the above two) | $108.82 | $50 | $200 | lognormal(log(108.82), 0.25)  Range: $47.83-$237.79  IQR: $91.76-$128.12 |  |

# Appendix 5. Model input values on treatment outcome

| **Surveillance arm** | **Bacterial infection** | **Culture positive** | **Clinical condition** | **Antibiotic change** | **Mortality of baseline scenario**  **(deterministic)** | **Number of hospitalisation days under baseline scenario (deterministic)** | **Justification** |
| --- | --- | --- | --- | --- | --- | --- | --- |
| Enhanced | yes | yes | not considered | Step-down | 20% | 7 | Set as baseline value |
| Enhanced | yes | yes | not considered | Step-up | 25% | 10.5 | The reason for higher mortality was that stepping up implied  that the empirical antibiotic treatment does not cover the causative organism (i.e. resistant to the treatment) |
| Enhanced | yes | yes | not considered | No change | 25% | 7 |  |
| Enhanced | yes | no | gets worse | Step up | 25% | 7 |  |
| Enhanced | yes | no | gets worse | No change | 37.5% | 10.5 | Higher mortality no change in treatment implied possibly higher probably of not have the antibiotic treatment covering the causative organism |
| Enhanced | yes | no | improves | Step-down | 8.3% | 2 | As clinical condition improved, mortality would be reduced |
| Enhanced | yes | no | improves | No change | 8.3% | 2 | As clinical condition improved, mortality would be reduced |
| Enhanced | yes | no | no change | Step up | 8.3% | 2 | Step-up would mean higher probability of covering the causative organism and clinical condition remains the same, hence mortality is the same as the above two conditions. |
| Enhanced | yes | no | no change | No change | 12.5% | 3.5 | The clinical condition remains the same, but no change in antibiotic would mean lower probability of covering the causative pathogen compared to stepping-up antibiotic treatment. Hence higher mortality compared to the above three conditions. |
| Enhanced | no | yes | not considered | Step-down | 10% | 3.5 | Half of the mortality of the corresponding “true bacterial infections” cases above, as in these cases there’s “no bacterial infection”. |
| Enhanced | no | yes | not considered | Step-up | 12.5% | 5 | Half of the mortality of the corresponding “true bacterial infections” cases above, as in these cases there’s “no bacterial infection”. |
| Enhanced | no | yes | not considered | No change | 12.5% | 3.5 | Half of the mortality of the corresponding “true bacterial infections” cases above, as in these cases there’s “no bacterial infection”. |
| Enhanced | no | no | gets worse | Step up | 12.5% | 3.5 | Half of the mortality of the corresponding “true bacterial infections” cases above, as in these cases there’s “no bacterial infection”. |
| Enhanced | no | no | gets worse | No change | 18.8% | 5 | Half of the mortality of the corresponding “true bacterial infections” cases above, as in these cases there’s “no bacterial infection”. |
| Enhanced | no | no | improves | Step-down | 4.2% | 1 | Half of the mortality of the corresponding “true bacterial infections” cases above, as in these cases there’s “no bacterial infection”. |
| Enhanced | no | no | improves | No change | 4.2% | 1 | Half of the mortality of the corresponding “true bacterial infections” cases above, as in these cases there’s “no bacterial infection”. |
| Enhanced | no | no | no change | Step up | 4.2% | 1 | Half of the mortality of the corresponding “true bacterial infections” cases above, as in these cases there’s “no bacterial infection”. |
| Enhanced | no | no | no change | No change | 6.3% | 2 | Half of the mortality of the corresponding “true bacterial infections” cases above, as in these cases there’s “no bacterial infection”. |
| No microbiological testing | yes | not done | gets worse | Step up | 31.3% | 9 | Higher mortality compared to “culture negative; gets worse; step-up” because “culture negative” implies low bacterial count. |
| No microbiological testing | yes | not done | gets worse | No change | 50% | 14 | Higher mortality compared to “culture negative; gets worse; step-up” because “culture negative” implies low bacterial count, and no change in antibiotic in this case implied higher probability not covering the causative organism compared to “step-up”. |
| No microbiological testing | yes | not done | improves | Step-down | 37.5% | 10.5 | Higher mortality compared to “culture negative; gets worse; step-up” because “culture negative” implies low bacterial count; whereas in this case step-down without culture support would have the risk of lowering the chance of treatment not covering the causative organism, |
| No microbiological testing | yes | not done | improves | No change | 31.3% | 9 | Higher mortality compared to “culture negative; gets worse; step-up” because “culture negative” implies low bacterial count, and no change in antibiotic in this case implied higher probability not covering the causative organism compared to “step-up”. However, lower mortality compared to “no microbiological testing; gets worse; no change” as clinical condition improves. |
| No microbiological testing | yes | not done | unchanged | Step up | 31.3% | 9 | Higher mortality compared to “culture negative; gets worse; step-up” because “culture negative” implies low bacterial count. The same mortality compared to “no microbiological testing; gets worse; step up” as stepping up increase the chance of antibiotic treatment covering the causative organism. |
| No microbiological testing | yes | not done | unchanged | No change | 37.5% | 10.5 | Higher mortality compared to “culture negative; gets worse; step-up” because “culture negative” implies low bacterial count. Higher mortality compared to “no microbiological testing; clinical confition unchanged; step-up” because no change (compared to step-up) has lower chance of antibiotic treatment covering the causative organism. |
| No microbiological testing | no | not done | gets worse | Step up | 12.5% | 3.5 | The same mortality as the corresponding “true bacterial infections and culture negative” cases above, as in these cases there’s “no bacterial infection”. |
| No microbiological testing | no | not done | gets worse | No change | 18.8% | 5 | The same mortality as the corresponding “true bacterial infections and culture negative” cases above, as in these cases there’s “no bacterial infection”. |
| No microbiological testing | no | not done | improves | Step-down | 4.2% | 1 | The same mortality as the corresponding “true bacterial infections and culture negative” cases above, as in these cases there’s “no bacterial infection”. |
| No microbiological testing | no | not done | improves | No change | 4.2% | 1 | The same mortality as the corresponding “true bacterial infections and culture negative” cases above, as in these cases there’s “no bacterial infection”. |
| No microbiological testing | no | not done | unchanged | Step up | 4.2% | 1 | The same mortality as the corresponding “true bacterial infections and culture negative” cases above, as in these cases there’s “no bacterial infection”. |
| No microbiological testing | no | not done | unchanged | No change | 6.3% | 2 | The same mortality as the corresponding “true bacterial infections and culture negative” cases above, as in these cases there’s “no bacterial infection”. |

# Appendix 6. Estimated incremental costs and disability-adjusted life years (DALYs) averted per 1,000 hospitalised paediatric patients from maintaining a microbiology laboratory compared to no microbiological testing under the baseline scenario. Costs are in 2022 US dollars.

|  | **An active laboratory** | | **No active microbiological testing** | |  |  |
| --- | --- | --- | --- | --- | --- | --- |
| **Empirical antibiotic therapy** | **Costs per 1,000 patients ($)** | **DALY per 1,000 patients** | **Costs per 1,000 patients ($)** | **DALY per 1,000 patients** | **Incremental costs ($)** | **DALY averted** |
| Ceftriaxone + gentamicin | 589,041·70 | 13,473 | 612,149·30 | 19,139 | -23,107.60 | 5,666 |
| Ampicillin + gentamicin | 596,101·20 | 13,487 | 723,567·80 | 18,809 | -127,466.60 | 5,322 |
| Meropenem + vancomycin | 650,906·50 | 12,614 | 577,225·70 | 19,375 | 73,680.85 | 6,761 |

# Appendix 7. Results of sensitivity analyses assuming two sets of blood samples (four bottles) for hospitalised adult patients with suspected primary bacteraemia.

|  | **An active laboratory** | | **No active microbiological testing** | |  |  |
| --- | --- | --- | --- | --- | --- | --- |
| **Empirical antibiotic therapy** | **Costs per 1,000 patients ($)** | **DALY per 1,000 patients** | **Costs per 1,000 patients ($)** | **DALY per 1,000 patients** | **Incremental costs ($) [IQR]** | **DALY averted** |
| Ceftriaxone + gentamicin | 622,232·10 | 2,636 | 672643·00 | 4,103 | -50,410·91 | 1,468 |
| Ampicillin + gentamicin | 636,638·20 | 2,640 | 815,587·20 | 4,030 | -178,949·00 | 1,390 |
| Meropenem + vancomycin | 816,600·20 | 2,479 | 794,267·20 | 4,156 | 22,333·01 | 1,677 |

# Appendix 8. Results of sensitivity analyses varying the cost of maintaining an active microbiology laboratory service per sample performed for hospitalised adult patients with suspected primary bacteraemia.


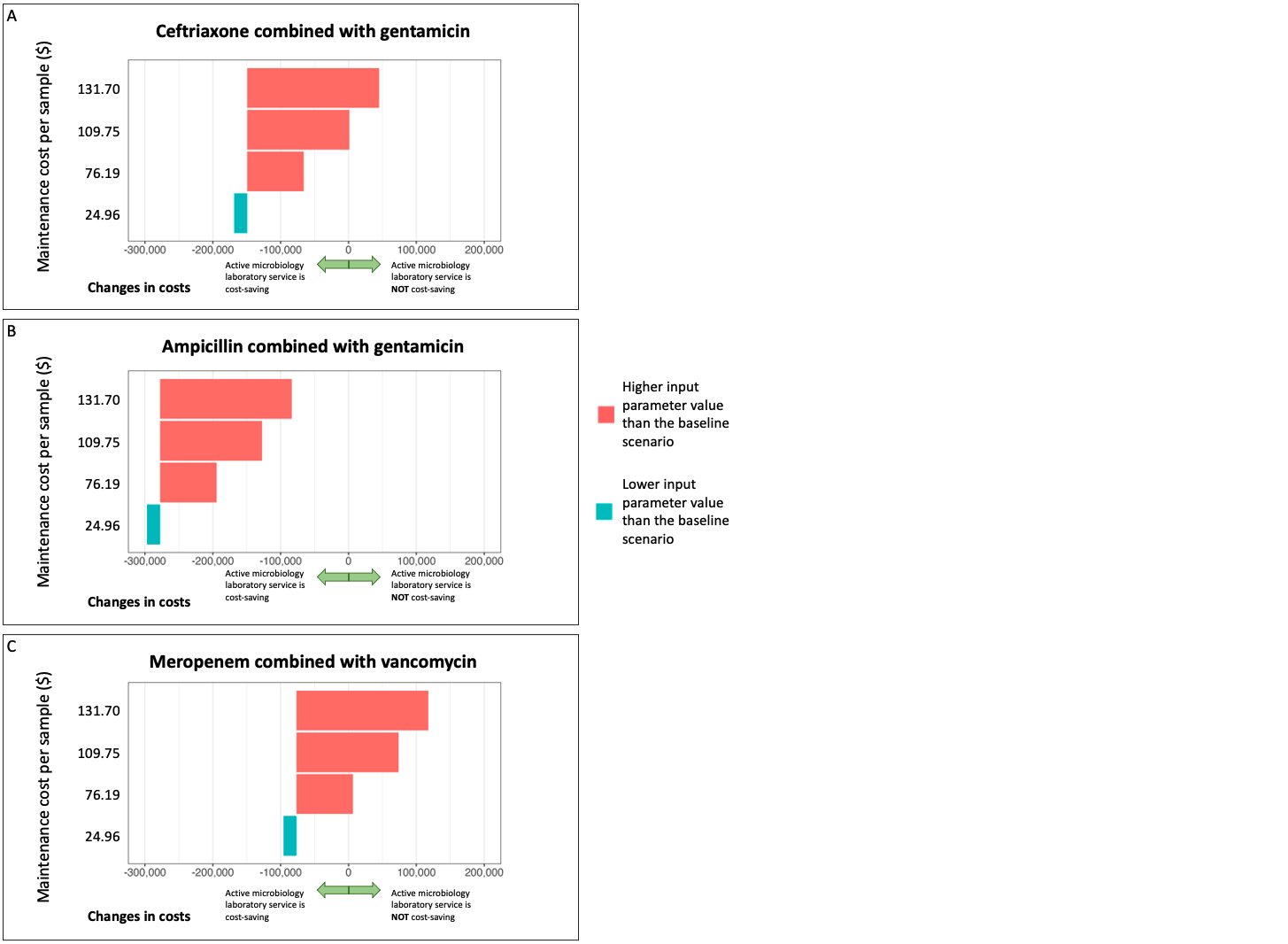

Supplement: Appendix [file mmc1.docx]
