# Supplementary material for: Cost-effectiveness of maintaining an active hospital microbiology laboratory service in Timor-Leste
Source: Lancet Reg Health Southeast Asia. 2025 Apr 30;36:100582. doi: 10.1016/j.lansea.2025.100582 (PMC12076793; doi:10.1016/j.lansea.2025.100582)
Supplement: Abstract in Tetum [file mmc2.docx]

**Evaluasaun kustu-effeitividade husi mantein servisu laboratoriu mikrobiolojia ativu ba nasaun ho rendimentu ki’ik no mediu sira: Uza Timor-Leste hanesan ezemplu**

Manutensaun laboratóriu mikrobiolojia ospitál ativu se fo dalan atu bele halo tratamentu antibiótiku definitivu ba infeksaun bakteriál iha tempu badak. Ho ida ne’e ita bele espera atu hadiak liu tan rezultadu pasiente no hamenus durasaun tempu baixa iha ospitál. Maibé, ospitál barak iha nasaun sira ho rendimentu ki'ik no médiu, laiha asesu ba servisu mikrobiolojia, no servisu mikrobiolojia ho kustu-efetividade ativu nian la klaru.

**Métodolojia**

Ami dezenvolve ona modelu ai-hun (skematiku) desizaun nian no hala'o ona análize kustu-efetividade atu determina karik manutensaun servisu laboratóriu mikrobiolojia ativu ida ne’ebe kustu-efetivu iha Timor-Leste–nasaun ida ho rendimentu médiu ki'ik. Modelu ne'e dezenvolve bazeia ba dadus mikrobiolojia lokál, kustu tratamentu pasiente lokál, rezultadu entrevista ho espesialista nian no dadus husi revizaun literatura sira.

**Rezultadu sira**

Kustu kuidadu pasiente média sei redúz to’o $165,469 (IQR: $134,834-200,902) ba kada pasiente ospitalizadu (baixa) nain 1,000 ho suspeita infeksaun raan, kompara se karik la iha laboratoriu mikrobiolojia ativu. Manutensaun laboratóriu mikrobiolojia ativu ida, tuir estimasaun, redúz nivel mortallidade entre 34 no 51 kada pasiente ospitalizadu 1,000. Rezultadu hanesan mos hetan husi análize sensitividade maneira oioin.

Tan ne’e, ami nia rezultadu sira indika katak iha probabilidade aas husi mantein laboratóriu mikrobiolojia ativu ida intervensaun kustu-efetivu ida, ne'ebé sei hadiak rezultadu pasiente no redúz kustu netu (tanba redúz admisaun kuidadu intensivu), se kompara fali ho laiha teste mikrobiolojia, liuliu ba pasiente pediátriku ospitalizadu sira ho suspeita bakteremia primária.

**Interpretasaun**

Ami nia rezultadu sira ne’e, indika katak investimentu iha manutensaun no espansaun kapasidade diagnóstika lokál nian posivelmente rezulta kustu-efetivu iha ambiente ho rekursu limitadu.
